# Error in Byline

**DOI:** 10.1001/jamanetworkopen.2026.40703

**Published:** 2026-10-02

The Original Investigation titled “Age at Menopause and Brain Atrophy Among Older Women,”^1^ published on August 25, 2026, was corrected to fix an error in Dr Dunietz’s name in the byline.
